# Pandemic risk: how large are the expected losses?

**DOI:** 10.2471/BLT.17.199588

**Published:** 2017-12-05

**Authors:** Victoria Y Fan, Dean T Jamison, Lawrence H Summers

**Affiliations:** a Office of Public Health Studies, Myron B Thompson School of Social Work, University of Hawai‘i at Mānoa, 1960 East-West Road, Honolulu, HI 96822, United States of America (USA).; b Institute for Global Health Sciences, University of California, San Francisco, San Francisco, USA.; c Harvard Kennedy School, Harvard University, Cambridge, USA.

## Abstract

There is an unmet need for greater investment in preparedness against major epidemics and pandemics. The arguments in favour of such investment have been largely based on estimates of the losses in national incomes that might occur as the result of a major epidemic or pandemic. Recently, we extended the estimate to include the valuation of the lives lost as a result of pandemic-related increases in mortality. This produced markedly higher estimates of the full value of loss that might occur as the result of a future pandemic. We parametrized an exceedance probability function for a global influenza pandemic and estimated that the expected number of influenza-pandemic-related deaths is about 720 000 per year. We calculated that the expected annual losses from pandemic risk to be about 500 billion United States dollars – or 0.6% of global income – per year. This estimate falls within – but towards the lower end of – the Intergovernmental Panel on Climate Change’s estimates of the value of the losses from global warming, which range from 0.2% to 2% of global income. The estimated percentage of annual national income represented by the expected value of losses varied by country income grouping: from a little over 0.3% in high-income countries to 1.6% in lower-middle-income countries. Most of the losses from influenza pandemics come from rare, severe events.

## Résumé

Il est nécessaire d'investir davantage dans la préparation contre les grandes épidémies et les pandémies. Les arguments en faveur de cet investissement s'appuient en grande partie sur les estimations des pertes au niveau du revenu national que pourrait entraîner une grande épidémie ou une pandémie. Récemment, nous avons élargi ces estimations pour y inclure la valeur des pertes faisant suite à des hausses de mortalité dues à des pandémies. Cela a donné des estimations nettement plus élevées de la valeur totale de la perte que pourrait occasionner une future pandémie. Nous avons paramétré une fonction de probabilité de dépassement pour une pandémie mondiale de grippe et avons estimé que le nombre escompté de décès dus à cette pandémie de grippe était d'environ 720 000 par an. Nous avons calculé que les pertes annuelles découlant du risque de pandémie représentaient environ 500 milliards de dollars des États-Unis, soit 0,6% du revenu mondial par an. Cette estimation rejoint (dans la fourchette inférieure) celles du Groupe d'experts intergouvernemental sur l'évolution du climat quant à la valeur des pertes dues au réchauffement de la planète, qui vont de 0,2% à 2% du revenu mondial. Le pourcentage estimé du revenu national annuel représenté par la valeur escomptée des pertes variait selon la catégorie de revenu des pays: d'un peu plus de 0,3% dans les pays à revenu élevé à 1,6% dans les pays à revenu intermédiaire-tranche inférieure. La plupart des pertes découlant de pandémies de grippe sont dues à des événements rares et graves.

## Resumen

Hay una necesidad no satisfecha de invertir más en la preparación para grandes epidemias y pandemias. Los argumentos a favor de dicha inversión se basan, en gran parte, en las estimaciones de las pérdidas en los ingresos nacionales que podrían darse como resultado de una gran epidemia o pandemia. Recientemente, ampliamos el cálculo para incluir la valoración de las vidas perdidas como resultado del aumento de la mortalidad relacionado con la pandemia. Esto dio como resultado unas estimaciones notablemente más altas del valor de la pérdida que podría resultar de una futura pandemia. Hemos parametrizado una función de probabilidad de excedencia para una pandemia de gripe mundial y estimado que el número esperado de muertes causadas por una pandemia de gripe es de aproximadamente 720 000 por año. Calculamos que las pérdidas anuales esperadas del riesgo de pandemia son de unos 500 000 millones de dólares estadounidenses, o el 0,6 % de los ingresos mundiales, por año. Esta estimación se encuentra dentro, pero cerca del mínimo, de las estimaciones del Panel Intergubernamental del Cambio Climático sobre el valor de las pérdidas por el calentamiento global, que oscilan entre el 0,2 % y el 2 % de los ingresos globales. El porcentaje estimado de los ingresos nacionales anuales representado por el valor esperado de las pérdidas varió según la agrupación de ingresos del país: de poco más del 0,3 % en los países con ingresos altos al 1,6 % en los países con ingresos medios o bajos. La mayoría de las pérdidas por pandemias de gripe provienen de casos raros y severos.

## ملخص

يوجد عجز في تلبية الحاجة إلى زيادة الاستثمار الذي يهدف إلى التأهب لمواجهة الأوبئة والأمراض الرئيسية واسعة الانتشار. وقد استندت الحجج المؤيدة لهذا الاستثمار بشكل كبير إلى تقديرات الخسائر في الدخل القومي والتي قد تقع نتيجة للأوبئة أو الأمراض الرئيسية واسعة الانتشار. واتسع نطاق تقديراتنا ليشمل تقييمًا لما نتج عن ارتفاع أعداد الوفيات المرتبطة بالأوبئة من خسائر في الأرواح. وقد نتج عن ذلك ارتفاع ملحوظ في تقديرات القيمة الكاملة للخسائر التي قد تقع نتيجة ظهور وباء مستقبلي. وقد قمنا بوضع معاملات قياسية لدالة احتمالات التجاوز فيما يتعلق بوباء الإنفلونزا المتفشي على مستوى العالم، وأشارت التقديرات إلى أن العدد المتوقع للوفيات الناجمة عن تفشي وباء الإنفلونزا يبلغ حوالي 720 000 نسمة في العام. وأشارت التوقعات، وفقًا لحساباتنا، إلى أن الأمراض المتفشية تهدد بوقوع خسائر سنوية تبلغ حوالي 500 مليار دولار أمريكي – أو 0.6% من الدخل العالمي – في العام الواحد. ويندرج هذا التقدير – الذي يعبر عن أقل القيم – ضمن ما توصلت إليه الهيئة الحكومية الدولية المعنية بتغير المناخ من تقديرات لقيمة الخسائر الناجمة عن الاحتباس الحراري، والتي تتراوح نسبتها بين 0.2% و2% من الدخل العالمي. إن النسبة المئوية المقدرة للدخل القومي السنوي والتي تمثلها القيمة المتوقعة للخسائر تتباين وفقًا لتصنيف الدول حسب الدخل: من نسبة تزيد قليلاً عن 0.3% في الدول عالية الدخل إلى نسبة 1.6% في الشريحة الأدنى من الدول متوسطة الدخل. وتقع معظم الخسائر الناجمة عن أوبئة الإنفلونزا نتيجة وجود حالات مرضية نادرة وحادة.

## 摘要

在应对重大流行病和大流行病方面，需要更多的投资。支持此类投资主要是基于对国民收入损失的估计，这些损失可能投入到主要流行或大流行病。最近，我们扩大了估计范围，包括因大流行病相关导致的死亡率增加而丧失的生命估值。这大大提高了对可能发生的损失价值全额的估计，这可能用于未来大流行病。我们对全球流感大流行的超过概率函数进行了参数化，并估计流感大流行导致的相关死亡人数每年约为 72 万人。我们计算出，每年的流感大流行损失预计将达到约 5000 亿美元——或占每年全球收入的 0.6%。这一估计属于政府间气候变化专门委员会对全球变暖造成的损失估计，但接近于较低端，全球变暖造成的损失占全球收入的 0.2% 到 2%。按国家收入分组计算的损失预期值所代表的年度国民收入的百分比：从高收入国家的略高于 0.3% 到低中等收入国家的 1.6%。流感大流行造成的大部分损失都来自罕见的严重事件。

## Резюме

Требуется увеличение инвестиций в подготовку к борьбе с крупными эпидемиями и пандемиями. Аргументы в пользу таких инвестиций в значительной степени основаны на оценках потерь в национальном доходе, которые могут возникнуть в результате крупной эпидемии или пандемии. Недавно авторы расширили эту оценку, включив в нее количество людей, погибших в результате увеличения смертности, связанной с пандемией. Это привело к более высоким оценкам полного ущерба, который может возникнуть в результате будущей пандемии. Авторы параметризовали функцию вероятности превышения смертности для глобальной пандемии гриппа и подсчитали, что прогнозируемое число смертей от гриппа и пандемии составляет около 720 000 в год. Мы подсчитали, что ожидаемые ежегодные потери из-за риска пандемии составляют около 500 млрд долларов США (или 0,6% мирового дохода) в год. Эта цифра находится в пределах оценки (ближе к нижней границе), полученной Межправительственной группой экспертов по изменению климата для оценки риска глобального изменения климата, которая составляет от 0,2 до 2% от глобального дохода. Предполагаемый процент годового национального дохода, представленный ожидаемой величиной потерь, варьировался по группам стран в зависимости от уровня дохода: от немногим более 0,3% в странах с высоким уровнем доходов до 1,6% в странах с низкими и средним доходом. Большинство потерь от пандемии гриппа происходят по причине редких тяжелых явлений.

## Introduction

Few doubt that major epidemics and pandemics will strike again and few would argue that the world is adequately prepared. Since the 2013–2016 Ebola virus disease outbreak in western Africa, the United States National Academy of Medicine^1^ and several other groups^2^^–^^4^ have pointed to gaps, and the need for greater investment, in preparation against epidemics and pandemics, of Ebola virus disease and other infectious diseases. Attempts to justify greater investment have mostly been based on estimates of the industrial and macroeconomic losses attributable to influenza pandemics.^5^^–^^11^ We have recently extended the loss assessment to include a valuation of the lives lost as a result of the increases in mortality resulting from influenza-pandemic risk.^12^ The inclusion of such a valuation increased the estimated loss attributable to modelled pandemic risk several fold. Below, we discuss our method and summarize our findings. Box 1 presents the definition of several of the terms we are using in this paper.

Box 1 **Definition of terms used in this article**

**Loss**

The consequences of a pandemic, in terms of lost income or lost lives.

**Costs**

The expenditures made to prepare for – or recover from – a pandemic.

**Pandemic severity**

Excess death attributable to a given influenza pandemic (expressed in this paper in standardized mortality units or SMUs - a unit of 1 per 10 000 per year).

**Pandemic risk**

The estimated probabilities that, in any given year, pandemics of varying degrees of severity will occur.

**Expected annual losses**

Defined in the probabilistic sense as the sum, across severities, of the losses associated with a pandemic of any given severity multiplied by the probability that a pandemic of that severity will occur in the coming year.

Note: Much of this nomenclature accords with that of the insurance industry.

## Valuing lives

Most previous economic studies on global influenza pandemics have focused on income losses, through reductions in the size of the labour force and productivity, increases in absenteeism and, importantly, as the result of individual and social measures that interrupt transmission, but disrupt economic activity. While measures such as the per-capita gross national income include the effect of pandemics on income, they also exclude the value of changes in mortality risk to individuals. If, in assessments of investments in pandemic preparedness and mitigation, we neglect this dimension of loss, we will underestimate the value of such investments, relative to alternative uses of public finances.

The broader approach that we recently applied, to the assessment of economic losses attributable to pandemic influenza, factors in the intrinsic loss associated with increases in mortality.^12^ In effect, this approach assigns a dollar value to small changes in mortality probabilities, using values derived from empirical studies of how individuals and societies actually value changes in mortality risk.^13^^–^^16^ This approach has already been employed extensively in environmental economics^13^^,^^14^ and has also been used in global health, by *The Lancet* Commission on Investing in Health.^15^^,^^16^

### Past literature

#### Economic losses from influenza

We searched Google Scholar and PubMed® for studies on the economic losses from influenza. Almost all of the previous studies examined economic losses in terms of income and ignored the value of, and the loss associated with, mortality risk. The World Bank, for example, generated estimates of global income losses under different influenza pandemic scenarios.^10^^,^^11^ It found that a pandemic of the same severity as the 1918 influenza pandemic might reduce global gross domestic product by about 5% and that the disruptive effects of avoiding infection would account for about 60% of that reduction. Another study of the consequences of a range of pandemic severities included an extremely severe scenario that would lead to income losses of over 12% of gross national income worldwide, including losses of over 50% of the gross national incomes of lower-income countries.^5^ We found other integrative estimates of the magnitude of pandemic risk in two partially proprietary sources.^17^^,^^18^

Several studies have examined specific dimensions of the economic impacts of annual influenza, such as direct costs, e.g. medical and hospitalizations costs, and indirect costs, e.g. lost earnings due to illness and productivity costs. There are examples of such studies based in the Americas,^6^^,^^7^^,^^19^^–^^22^ Asia^8^^,^^23^ and Europe.^24^^,^^25^ Other models have added an estimated value of the intrinsic undesirability of nonfatal illness or of pandemic fear, as seen in the population response to severe acute respiratory syndrome in Asia.^8^ Media coverage may also lead populations to overreact to mild pandemics.^9^

We found only two articles that included estimates of the loss from the elevated mortality associated with influenza pandemics.^8^^,^^19^ Of the 10 studies included in a recent systematic literature review on the costs of influenza,^26^ only one^19^ took account of the value of mortality risks.

#### Value of a statistical life

One strand of economic research has examined the intrinsic value of mortality risks, which is commonly expressed as the so-called value of a statistical life. This value is derived either from questionnaires that canvass how much compensation an individual would demand, to accept a small increase in the probability of their death,^1^ or from quantitative studies of the labour market that investigate the trade-offs between small fatality risks and income.^2^^,^^27^

Beyond influenza, the value of mortality risks has been included in estimating the costs of vaccine-preventable diseases^28^ and in evaluating the economic burdens posed by rheumatic heart disease.^29^ Far more studies have assessed the burden of specific environmental risk factors.^13^^,^^14^

The value of a statistical life, which is sometimes expressed as the value of a standardized mortality unit (SMU), i.e. an increase in the annual risk of death of 1 in 10 000, varies by both the age and income of the individual involved.^15^^,^^16^^,^^27^ In general, the value of mortality is elastic to age and to income, i.e. younger individuals place a higher value on mortality than older individuals, and higher-income individuals generally value mortality more than lower-income individuals. The main findings of our recent study appeared consistent when, in robustness and sensitivity checks, we used estimates that were unconditional on age and estimates with varying income elasticity with respect to the value of mortality.^12^

## Expected-loss framework

Given the uncertain nature of an influenza pandemic, in terms of both when it may occur and how large the mortality risks will be, we applied an expected-loss framework that accounts for the uncertainty over a long period of time.^7^ An expected-loss framework incorporates information on the risk of an uncertain event, e.g. a pandemic, with information on the severity or value of that event, e.g. the increase in mortality. Although it has been estimated that the 2013–2016 Ebola virus disease outbreak led to about 11 300 deaths,^30^ the death toll from a severe influenza pandemic might be 2500 times higher than this.^12^ In any given year, however, the risk of a severe influenza pandemic is much smaller than that of an Ebola epidemic. The use of an expected-loss framework allows policy-makers to compare the expected losses associated with events with relatively high annual probability, but low mortality, e.g. an Ebola outbreak, with those of events with relatively low probability but high mortality, e.g. the 1918 influenza pandemic.

### Exceedance probability function

Expected-loss frameworks are commonly used, by actuaries in the insurance industry, to calculate the size of premiums, e.g. for flood or health insurance. To value the consequences of uncertain events appropriately, the insurance industry estimates so-called exceedance probability functions. These functions generate estimates of the probability that, over a specified time frame, losses from an uncertain event, e.g. an influenza pandemic, would exceed any specified level. For our analysis, we developed an exceedance probability function for a global influenza pandemic. To parameterize the function, we turned to historical data on global influenza pandemics since the 1700s.^31^^–^^34^ Six pandemics in this period led to excess mortality rates ranging between 0.03% and 0.08% of world population. In 2017, this range would be the equivalent of between 2 million and 6 million excess deaths globally. A modelling exercise for the insurance industry concluded that the annual risk of an influenza outbreak on the scale of the 1918 pandemic lies between 0.5% and 1.0%.^18^ For more severe pandemics, we fitted a parametrized exceedance probability function to modelled data that had been previously reported.^18^^,^^35^

#### Model calibration

Following common practice in the insurance industry, we defined risk, r(*s*), in terms of the annual probability of a pandemic having a severity exceeding *s* SMUs and the return time for *s* as the expected number of years before a pandemic of at least severity *s* will occur. If t(*s*) is the return time, then t(*s*) = r(*s*)^−1^. For example, if the annual probability of a pandemic of severity at least *s* is 1%, then its return time will be 100 years.

If we had access to a function r(*s*) showing exceedance probability as a function of severity, our analysis could proceed using the expected value of severity of all pandemics. Because r(*s*) is the complementary cumulative of the density for *s*, we would have expected value of:

*Expected value of s* =∫^∞^_0_*r(s) d(s)* (1)

We calibrated this model using historical estimates of the frequency and severity of influenza pandemics, which we obtained from our literature search on PubMed® and Google Scholar. For mortality data relating to the 1918 influenza pandemic, we also searched the libraries at Harvard University and the University of Hawai‘i for historical documents and life tables. Studies were restricted to those with abstracts in English.

Like other economic studies of pandemic influenza, we identified two main influenza pandemic scenarios in terms of aggregate mortality: moderate and severe. Our review classified the 1918 pandemic as severe. As the world population in 1918 was about 1830 million and historical data indicate that there were at least 20 million pandemic-related deaths in that year, the excess death rate associated with the pandemic was at least 1.1%. A closer examination of the data from India indicate that the true global rate was probably far higher than 1.1%, the pandemic led to 14 million deaths in India^36^^–^^38^ and it seems implausible that India accounted for 73% of all of the pandemic-related deaths at a time when it had 18% of the world population. However, to be conservative, we estimated an expected annual excess mortality rate of 0.93 SMUs. In the corresponding model for moderate pandemics, we used a global expected excess mortality rate of 0.05 SMUs, as seen in historical moderate pandemics.^39^

Our calibration pointed to a very fat-tailed distribution.^12^ Thus, compared with an exponential function, the hyperbolic family of complementary cumulative distributions provided more natural candidates for r(*s*). We parameterized the hyperbolic function in terms of its expectation and the fatness of its tail.^40^ Thus:

r(*s*) = [1 + *m*(1 – *f*)*s*] ^– [1 + 1/(1 –^ *^f^*^)]^ (2)

where *f* indicates the fatness of the tail, with smaller values implying a fatter tail. We estimate a value of *f* of -2. It had previously been estimated that, in 2015, a 1918-type pandemic would have killed 21 million to 33 million people, with a return time of 100–200 years.^35^ Our models produced similar values. Recent estimates of influenza and pneumonia mortality^18^^,^^31^ are also consistent with our all-cause mortality estimates. Our estimates are based on assumptions that are probably quite conservative. Substantially greater severities and likelihoods have been discussed elsewhere.^5^^,^^35^^,^^41^^,^^42^

## Mortality-inclusive value of losses

We used our estimated exceedance probability function and empirically estimated values for small changes in mortality risk to calculate the expected i.e. mortality-inclusive, value of losses associated with a moderate or severe influenza pandemic. At 2013 values, the expected losses for 2015 amounted to about 500 billion United States dollars (US$), i.e. about 0.6% of global income, per year.^12^ The estimated proportion of annual national income represented by the losses varied according to country income grouping, from a little over 0.3% in high-income countries to 1.6% in lower-middle-income countries (Table 1).

**Table 1:** Mortality and economic losses of influenza-pandemic risk, 2015

Variable**^a^**	Country income group				World
	Low	Lower-middle	Upper-middle	High	
Expected mortality (thousands of deaths/year)	120	390	180	28	720
Expected annual economic losses (% of GNI/year)^b^	1.1	1.6	1.0	0.3	0.6

GNI: gross national income.

^a^ Data are based on modelled risk of either a moderate or severe pandemic in 2015.^12^

^b^ Both loss of national income and intrinsic loss associated with elevated mortality.

The expected-loss framework distinguishes between the loss associated with a certain event that occurred, e.g. the mortality that occurred as a result of the 1918 influenza pandemic, and the expected loss associated with an uncertain event over a period of risk exposure. The expected loss combines both the risk of a moderate or severe pandemic and the losses from that event should the event occur. The expected-loss framework thus produces estimates of expected losses of an uncertain event, rather than actual losses of a certainly occurring event. We estimated the expected number of pandemic-related deaths to be about 720 000 per year. This level of mortality is on a similar scale to that attributable to other, more certain, causes of death, including other major infectious causes of death.^12^

Importantly, we concluded that most of the expected loss from influenza pandemics results from extreme events. Another effort to estimate exceedance probability functions indicated that, among all pathogens that can cause a pandemic, influenza virus was likely to be the predominant cause of pandemic-related mortality.^18^ The implication is clear: any efforts at pandemic preparedness need to be most strongly focused on influenza and on preparation for a severe scenario.

Our results present losses much higher than those found in studies limited to income losses. Income losses have been estimated to represent around 15% and 50% of the total economic losses associated with a severe pandemic and a mild pandemic, respectively.^5^^,^^11^ In previous studies, across modelled pandemics of all severities, mean income losses were estimated to be US$ 80 billion per year^5^^,^^11^, i.e. about 16% of our estimate of total pandemic-related costs.

## Climate change comparison

In terms of the percentage of global income, our estimate of total pandemic-related losses (0.6%) falls within the corresponding Intergovernmental Panel on Climate Change’s estimates of the costs of global warming (0.2–2.0%).^43^ However, the magnitude of future global warming and the associated economic losses are still uncertain.^44^^,^^45^ The same is true for future pandemics. Many of the hundreds of studies on the potential costs of climate change^46^ have been hampered by the wide variation in estimates of the so-called social cost of carbon.^47^ If this cost is set at about US$ 120 per tonne, the cost of the carbon dioxide emissions in 2013 would have been about 1% of global income.^46^^,^^48^ As in many previous attempts to estimate the economic losses associated with a pandemic, many previous attempts to estimate the social costs of carbon have focused on national income accounts, without any explicit valuation of the increases in mortality resulting from climate change. The mortality-associated costs of climate change may be relatively small, however, since the slowness of climate change should allow for compensatory human adaptation.

## Limitations

Our study had several limitations. First, we ignored the intrinsic undesirability of nonfatal illness and/or pandemic fear. Intense media coverage may lead populations to overreact to mild pandemics. Second, our estimates of future pandemic risk and severity, and the economic estimates based on these epidemiological estimates, are relatively crude partly because pandemics remain rare and uncertain events. Future modelling should lead to improved estimates over time. Third, the assignment of monetary value to small changes in mortality risk and, particularly the relationship between valuation of such risk and both individual income and age at death, remains controversial. However, the results of sensitivity analyses, in which we applied a range of assumptions on these parameters, indicated that our main findings were reasonably robust.

## Policy development

In addition to pathogens of pandemic potential, an expected-loss framework may also be applied usefully to malaria and other diseases that have fluctuating incidence. As cases of the disease become rarer as the result of effective interventions, malaria becomes less visible politically and financially, and policy-makers in some countries may have responded by reducing control efforts prematurely. Policy-makers, and the societies they serve, could benefit by using an expected-loss framework to estimate the losses associated with uncertain and rare events across the full range of potential outcome severities. This could lead to appropriate and beneficial adjustments to each policy-maker’s sense of risk and sense of value and to improved national policies on epidemic and pandemic preparedness. A recent United States National Academy of Medicine report argued that, given the risks we estimated, policy attention has fallen short.^49^^,^^50^ National efforts at pandemic preparedness have benefits beyond national borders. Some have therefore argued that, for the global good, resources for development assistance should be used to provide incentives for national investments and international collaborations in such preparedness.^3^
